# *ccdc80-l1* Is Involved in Axon Pathfinding of Zebrafish Motoneurons

**DOI:** 10.1371/journal.pone.0031851

**Published:** 2012-02-22

**Authors:** Chiara Brusegan, Anna Pistocchi, Andrea Frassine, Isabella Della Noce, Filippo Schepis, Franco Cotelli

**Affiliations:** 1 Dipartimento di Biologia, Università degli Studi di Milano, Milan, Italy; 2 Dipartimento di Medicina Rigenerativa, DiBiT, Istituto Scientifico San Raffaele, Milan, Italy; 3 Dipartimento di Medicina, Università di Modena e Reggio Emilia, Modena, Italy; VIB & Katholieke Universiteit Leuven, Belgium

## Abstract

Axon pathfinding is a subfield of neural development by which neurons send out axons to reach the correct targets. In particular, motoneurons extend their axons toward skeletal muscles, leading to spontaneous motor activity. In this study, we identified the zebrafish Ccdc80 and Ccdc80-like1 (Ccdc80-l1) proteins *in silico* on the basis of their high aminoacidic sequence identity with the human CCDC80 (Coiled-Coil Domain Containing 80). We focused on *ccdc80-l1* gene that is expressed in nervous and non-nervous tissues, in particular in territories correlated with axonal migration, such as adaxial cells and muscle pioneers. Loss of *ccdc80-l1* in zebrafish embryos induced motility issues, although somitogenesis and myogenesis were not impaired. Our results strongly suggest that *ccdc80-l1* is involved in axon guidance of primary and secondary motoneurons populations, but not in their proper formation. *ccdc80-l1* has a differential role as regards the development of ventral and dorsal motoneurons, and this is consistent with the asymmetric distribution of the transcript. The axonal migration defects observed in *ccdc80-l1* loss-of-function embryos are similar to the phenotype of several mutants with altered *Hedgehog* activity. Indeed, we reported that *ccdc80-l1* expression is positively regulated by the Hedgehog pathway in adaxial cells and muscle pioneers. These findings strongly indicate *ccdc80-l1* as a down-stream effector of the Hedgehog pathway.

## Introduction

The development of a functional vertebrate nervous system requires elaboration of a large number of diverse cell types. At embryonic stages, the nervous system is a complex network of growing axons, whose growth cones navigate in response to guidance cues. Among them, motoneuron axons migrate toward skeletal muscles, and form synaptic contacts [1]. Zebrafish embryos exhibit spontaneous contractions of the musculature ever since 18–19 hours post fertilization (hpf) when removed from their protective chorion [2]. These movements are due to the early-developing primary motoneurons (PMNs), that innervate the myotome with nonoverlapping arbors. In zebrafish, PMNs are present in each somitic hemisegment and are identified by their specific axonal pathway and soma position within the spinal cord: caudal primary motoneurons, middle primary motoneurons and rostral primary motoneurons (CaPs, MiPs and RoPs, respectively) [3], [4], [5]. PMNs extend their axons out of the spinal cord at about 16–17 hpf, following a common pathway: their growth cones project ventrally along the medial surface of the myotome and pause at the horizontal myoseptum, which separates dorsal and ventral myotomes. Here, they specifically interact with muscle pioneers [6], [7], a subset of two to six cells for each somite early differentiating into slow muscle fibers [8], [9]. CaPs are responsible for pioneering the common pathway before projecting the axons that innervate the ventral myotome [10]. Among PMNs, they show the largest and most extensive branching pattern [5]. MiPs sprout a collateral axon to innervate dorsal myotome, while the first ventral process extending along the common pathway is retracted by 48 hpf [7]. RoPs innervate the middle region of the muscle segment, sprouting laterally after pausing at the myoseptum [6]. Therefore, muscle pioneers represent a choice point from which motoneurons select their specific pathway, although the ablation of this cell population leads to abnormal motor axonal extension without altering the target choice [7]. Secondary motoneurons (SMNs) growth cones extend later from spinal cord, beginning at 22 hpf and following the paths pioneered by the primary axons [11], [12].

Axonal pathfinding is dependent on attractive and repulsive stimuli coming from both nervous and non-nervous surrounding tissues [1], [13]. For instance, *shh* induction by notochord and floorplate patterns both primary and secondary motoneurons [14]. Indeed, mutants lacking both the notochord and the floorplate (*cyc^−^;flh^−^*) [14] or mutants in the Hedgehog pathway, such as *smoothened* (*smo*), present disorganized, reduced or absent PMNs and axons. [15]. Moreover, in *sonic-you* mutants (*syu*) CaPs and MiPs axons run along the neural tube horizontally instead of ventrally and dorsally, while axons of the secondary motoneurons fail to branch and instead cease to extend or grow further ventrally in an abnormal pattern [16].

Also muscular tissues can pattern axonal migration: muscular adaxial cells are able to rescue motor axon defects in *diwanka* mutants, showing that this myotomal population plays a pivotal role in axonal migration [17]. Furthermore, molecules expressed in the somites such as the semaphorin proteins Z1b and Sema3A1 are involved in repelling axonal migration [10], [18]. On the contrary, *netrin-1a* is expressed by adaxial cells and muscle pioneers besides ventral spinal cord, and seems to guide axonal growth through a chemoattractive function [19]. The manipulation of the proper expression of these molecules, both knocking-down and inducing ectopic expression, induces axons to follow aberrant pathways, branch excessively or stall [17], [18], [19].

The *Coiled-Coil-Domain Containing 80* (*Ccdc80*) gene, also named *DRO1* in human (*Down-Regulated by Oncogene 1*), *URB* in mouse (*Up-Regulated in BRS-3 deficient mice*), *CL2* in rat (*Clone 2*), and *equarin* in chicken, has been recently suggested to be involved in different functions among vertebrates. *Ccdc80* was first isolated in mice, where it is up-regulated in adipose tissue of obese BRS-3-deficient animals [20]. Moreover, *Ccdc80* is highly expressed in mice white adipose tissue and its silencing inhibits adipocytes differentiation [21], suggesting a role in the regulation of body weight and energy metabolism. *Ccdc80* is also expressed in mouse developing cartilage, suggesting a role during skeletogenesis [22]

Human *CCDC80* is almost ubiquitously expressed, with the highest levels in heart and skeletal muscles [23], [24]. Furthermore, human *CCDC80* can be considered a potential tumor suppressor gene [25]. In fact, it is strikingly down-regulated in thyroid neoplastic cell lines and tissues, as well as in colon and pancreatic cancer cell lines and in most colorectal cancer specimens [26], while its ectopic expression in these cell lines results in substantial inhibition of growth properties.

The CCDC80 protein is highly conserved among vertebrates, and contains multiple signals of cellular compartmentalization and post-translational modifications. In particular, it has a N-terminus leader peptide for extracellular export and many nuclear localization signals [25]. In different studies, the CCDC80 protein has been identified in a N-glycosylated form and was suggested to be secreted. Rat, mouse and human CCDC80 show three P-DUDES domains (Procaryotes- *DRO1-URB-DRS-Equarin-SRPUL*) which in human are correlated with a tumor suppressor role [27].

*In silico* analysis using human CCDC80 sequence as a bait, led to the identification of three zebrafish homologs of CCDC80. Two homologs, that we named Ccdc80 and Ccdc80-like1 (Ccdc80-l1), showed high levels of aminoacid identity with the human CCDC80. We performed the molecular cloning of *ccdc80* and *ccdc80-l1* in zebrafish and analyzed their expression pattern during embryonic development. During somitogenesis *ccdc80* is expressed in the notochord (manuscript in preparation), while *ccdc80-l1* is expressed in muscle pioneers and adaxial cells. Both these regions are responsible for axon guidance, therefore we decided to investigate the role of *ccdc80* and *ccdc80-l1* in this process. While loss-of-*ccdc80-*function did not impair motoneurons development, we demonstrated the *ccdc80-l1* involvement in the proper axonal pathfinding, especially in ventral axons guidance. Indeed, *ccdc80-l1* knocked-down embryos exhibited motility issues although analysis on body musculature showed that somitogenesis and myogenesis occurred properly. Furthermore, the analysis of *ccdc80-l1* up-stream regulation revealed that the Hedgehog pathway modulates its expression in territories involved in axonal guidance.

## Materials and Methods

### Zebrafish lines and maintenance

Current italian national rules: no approval needs to be given for research on zebrafish embryos. Zebrafish were raised and maintained according to established techniques [28], approved by the veterinarian (OVSAC) and the animal use committee (IACUC) at the University of Oregon, in agreement with local and national sanitary regulations. Embryos were collected by natural spawning, staged according to Kimmel [29], and raised at 28°C in fish water (Instant Ocean, 0.1% methylene blue) in Petri dishes [30].

### Sequence analysis

Zebrafish chromosome 6 region hosting the *ccdc80-l1* gene was identified through *in silico* search of the ENSEMBL zebrafish assembly version 9 (Zv9) using human CCDC80 aminoacidic sequence as a bait. The alignments between aminoacid sequences were performed with the software program StrecherP. Analysis on synteny was performed with the program Genomicus version 57.01.

### RT-PCR

Total RNA from 11 samples (an average of 30 embryos per sample) corresponding to 9 different developmental stage embryos (2–4 cells, 64–1000 cells, 30% epiboly, 60%–70% epiboly, somitogenesis, 24 hpf and 72 hpf) and 2 adult organs (ovary and muscle) was extracted with the TOTALLY RNA isolation kit (Ambion), treated with RQ1 RNase-Free DNase (Promega) and oligo (dT)-reverse transcribed using Super- Script II RT (Invitrogen), according to manufacturers' instructions. The following primers were used for PCR reactions: *ccdc80-l1*_forward 5′- ACCACAATGGAGCAAACACA -3′ and *ccdc80-l1*_reverse 5′-GGTTTAGCTCTCCCCTTTGG -3′. PCR products were loaded and resolved onto 2% agarose gels.

### *In situ* hybridization and immunohistochemistry

Whole-mount *in situ* hybridization (WISH), was carried out as described [31] on embryos fixed for 2 hours in 4% paraformaldehyde in PBS, then rinsed with PBS-Tween (PBT), dehydrated in 100% methanol and stored at −20°C until processed [32]. Antisense riboprobes were previously *in vitro* labelled with modified nucleotides (digoxigenin, Roche). *myod* and *myog* probes were prepared as described by Schnapp and collegues [33]. *smyhc1* probe has been kindly provided by Ingham laboratory. The following primers were used for PCR reactions to clone the probes: *ccdc80-l1* sense 5′- ACCACAATGGAGCAAACACA -3′ and *ccdc80-l1* antisense 5′- GGTTTAGCTCTCCCCTTTGG -3′. For immunohistochemistry, embryos were fixed in 4% paraformaldehyde overnight at 4°C or 2 hours at RT, washed several times in PBT and blocked in 5% BSA in PBT for 1 hour at room temperature. Primary antibody incubation was done overnight at 4°C, followed by several washes in PBT and incubation of secondary antibody for 1 hour at room temperature. Primary antibodies are MF20 (mouse anti-sarcomeric) and 4D9 (mouse anti-engrailed/invected) purchased from Developmental Studies Hybridoma Bank, znp1 (mouse anti-*syt2b*) and zn-5 (mouse anti-*alcama*) purchased from Zebrafish International Resource Center (ZIRC). Secondary antibody is EnVision+ System- HRP Labelled Polymer anti-mouse (Dako). Images of embryos and sections were acquired using a microscope equipped with a digital camera with LAS Leica imaging software (Leica, Wetzlar, Germany). Images were processed using the Adobe Photoshop software. For histological sections, stained embryos were re-fixed in 4% paraformaldehyde, dehydrated and stored in methanol, wax embedded, and sectioned (5–8 µm).

### Injections

Injections were carried out on 1- to 2-cell-stage embryos (with Eppendorf FemtoJet Micromanipulator 5171); the dye tracer rhodamine dextran was co-injected as a control. To repress *ccdc80-l1* mRNA translation we designed an ATG-targeting morpholino, *ccdc80-l1*-MO: 5′- TTGTACCTGTAGATTTTTCATTGCA-3′ and a splice-site morpholino, *ccdc80-l1*-splice- 5′- TGATACAATACATACTATGAGGCGT -3′ (Gene Tools, LLC). As a negative control we injected a standard control morpholino oligonucleotide (ctrl-MO). Morpholinos were injected in 1× Danieau buffer (pH 7.6) as suggested by Nasevicius and Ekker [34]. For the *in vivo* test of the efficiency of *ccdc80-l1*-MO, 425 pg/embryo of the pCS2+-*ccdc80-l1*-GFP sensor plasmid have been injected alone or co-injected with 12 ng/embryo of *ccdc80-l1*-MO. The presence/absence of the GFP signal has been monitored under a fluorescent microscope starting from somitogenesis up to 48 hpf. *ccdc80-l1*-MO cDNA fragments inserted in the *BamHI* site were obtained using the following complementary oligos: *ccdc80-l1*-MO sense 5′- gatcTTGTACCTGTAGATTTTTCATTGCACA -3′ and *ccdc80-l1*-MO antisense 5′ – gatcTGTGCAATGAAAAATCTACAGGTACAA- 3′.

For the *in vivo* test of the specificity of morpholino-mediated knockdown, the rescue of morphants phenotype was obtained co-injecting 12 ng/embryo of *ccdc80-l1*-MO together with 400 pg/embryo of *ccdc80*-encoding mRNA.

Over-expression of *shh* was obtained microinjecting 300 pg/embryo of *shh* mRNA, kindly provided by Sordino laboratory.

### Statistical analysis

Statistical analysis was performed with Student's t-test using GraphPad PRISM version 5.0 (GraphPad, San Diego, California). A p value <0.001 indicates a statistically significant effect.

### Cyclopamine treatment

Embryos were exposed to 5 µM cyclopamine (purchased from SIGMA-ALDRICH) from 50% epiboly stage up to fixation in PFA at 15 somites stage. Cyclopamine was dissolved in embryo medium and 0.5% ethanol. Controls consisted of corresponding incubations in 0.05% ethanol in embryo medium.

## Results

### Identification of *Ccdc80* homologs in the genome of zebrafish

**Figure 1:**
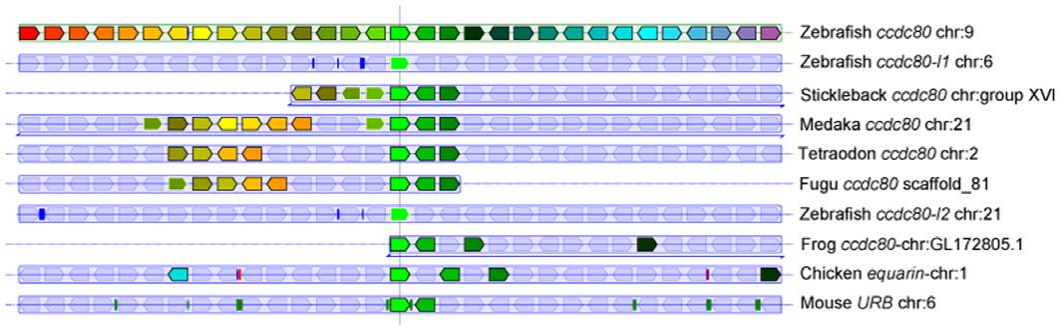
Analysis of chromosomal organization of the three *ccdc80* zebrafish homologs across vertebrates. Each *ccdc80* gene is shown as a reference locus. Genes annotated as paralogs (no surrounding line) or orthologs (with a surrounding line) by the Ensembl database share the same color, blue lines beneath individual tracks indicate that orientations of gene blocks and are inverted with respect to their genomic annotation. For zebrafish *ccdc80* (chr. 9), *ccdc80-l1* (chr. 6) and *ccdc80-l2* (chr. 21), only *ccdc80* shows notable synteny with other vertebrates. The figure was derived from the output of the Genomicus website (version 57.01).

Blast analysis of the ENSEMBL zebrafish assembly version 9 (Zv9) using human CCDC80 as a bait returned three positive hits, corresponding to three proteins encoded by genes on different chromosomes: the first on chromosome 9 (nucleotide position: 35,060,460-35,084,513) that we named *ccdc80*, the second on chromosome 6 (nucleotide position: 16,322,724-16,342,517) that we named *ccdc80-like1* (*ccdc80-l1*), and the third on chromosome 21 (nucleotide position: 18,662,129-18,669,986), that we named *ccdc80-like2* (*ccdc80-l2*). The alignment of the predicted protein sequences in zebrafish with the human CCDC80, revealed that Ccdc80 presented the highest degree of aminoacid identity with human CCDC80 (51.6%), while Ccdc80-l1 and Ccdc80-l2 presented less identity (44.4% and 27% respectively) (Table 1 and Fig. S1). We then performed alignments among the three zebrafish homologs: Ccdc80 shared the 51.4% of aminoacid identity with Ccdc80-l1 and the 30.4% with Ccdc80-l2 while Ccdc80-l1 and Ccdc80-l2 shared the 29.1% of identity (Table 1). Moreover, analysis of chromosomal organization of the three *ccdc80* zebrafish homologs across vertebrates revealed that only *ccdc80* is synthenic with other vertebrates (Fig. 1).

**Table 1:** Percentages of identity and similarity among human and zebrafish Ccdc80 homologs.

	Human CCDC80		Zebrafish Ccdc80		Zebrafish Ccdc80-like1		Zebrafish Ccdc80-like2	
	Identity	Similarity	Identity	Similarity	Identity	Similarity	Identity	Similarity
Zebrafish Ccdc80	51,6%	65,2%	/	/	51,4%	64,4%	30,4%	47,4%
Zebrafish Ccdc80-like1	44,4%	59,3%	51,4%	64,4%	/	/	29,1%	46,9%
Zebrafish Ccdc80-like2	27%	44,9%	30,4%	47,4%	29,1%	46,9%	/	/

The table shows the scores obtained after alignments between the aminoacidic sequences of zebrafish and human CCDC80 homologs. Alignments were performed with Stretcher-P tool.

### *ccdc80-l1* is expressed in muscle pioneers and adaxial cells of the zebrafish embryo

**Figure 2:**
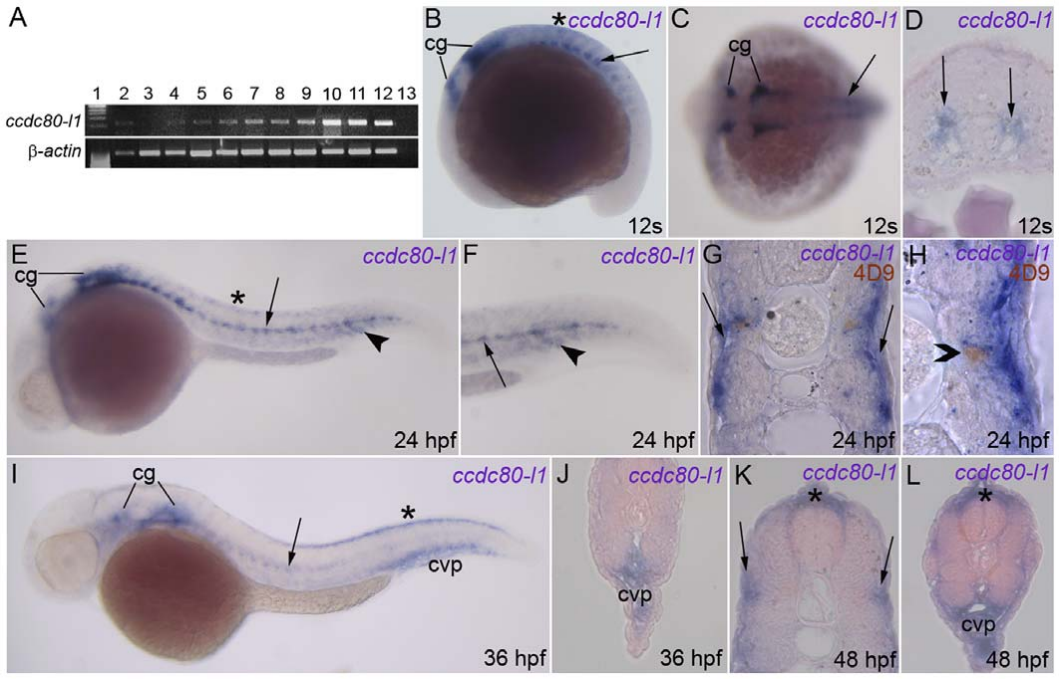
Expression of *ccdc80-l1* analyzed by RT-PCR and WISH. *(A)* RT-PCR performed on different embryonic stages and adult tissues; the expression of *ccdc80-l1* and *β-actin* are shown. Lanes are: ladder (lane 1), ovary (lane 2), 2–4 cells stage (lane 3), 64–1000 cells stage (lane 4), 30% epiboly (lane 5), 60–70% epiboly (lane 6), somitogenesis (lane 7), 24 hpf (lane 8), 30 hpf (lane 9), 48 hpf (lane 10), 72 hpf (lane 11), adult muscle (lane 12) and negative control (lane 13) in the absence of cDNA. *(B–J)* WISH performed on zebrafish embryos at several stage of development. *(B, C)* During somitogenesis *ccdc80-l1* was expressed by cranial ganglia (cg), dorsal dermis (asterisk), adaxial cells and muscle pioneers at the level of the horizontal myoseptum (arrow). *(D) ccdc80-l1* expression in a transverse section of the trunk of an embryo at 12 somites stage (arrows). *(E–H)* At 24 hpf, the hybridization signal was detectable in cranial ganglia (cg), dermis (asterisk), adaxial cells (arrow) and ventral somites (arrowhead). *(F)* Higher magnification of the tail at 24 hpf. *(G)* Transversal section of an embryo at 24 hpf. *(H)* Transversal section showing that at 24 hpf *ccdc80-l1* hydridization signal co-localized with the nuclear labeling of 4D9 antibody, corresponding to the engrailed-positive muscle pioneers population (open arrowhead). *(I, J)* At 36 hpf, the signal of *ccdc80-l1* probe was detected in cranial ganglia (cg), migrated adaxial cells (arrow), dorsal dermis (asterisk) and caudal vein plexus region (cvp). *(K, L)* At 48 hpf, *ccdc80-l1* was detected in dorsal dermis (asterisk), external adaxial cells (arrows in *K*) and caudal vein plexus region (cvp in *L*). *(B, E, F, I)* Lateral views; dorsal is up, anterior is left; *(C)* dorsal view, anterior is left; *(D,G, H, J–L)* transversal sections, dorsal is up.

Characterization of *ccdc80-l1* expression, using RT-PCR, revealed that *ccdc80-l1* transcript is present from the first stages of development up to 72 hpf, thus including maternal and zygotic transcription (Fig. 2A). *ccdc80-l1* is also expressed in the ovary and muscle of the adult zebrafish (Fig. 2A). During somitogenesis, the hybridization signal is restricted to the horizontal myoseptum (Fig. 2B–D). From this stage, *ccdc80-l1* expression is observed also in the cranial ganglia and dorsal dermis (Fig. 2B, 2C, 2E, 2I, 2K–L and data not shown). At 24 hpf, *ccdc80-l1* is detectable in a specific sub-population of migrating adaxial cells, that moves along the lateral axis towards the external somite [35] (Fig. 2E–G). Moreover, *ccdc80-l1* is expressed in muscle pioneers, as shown by the co-localization between *ccdc80-l1* and engrailed [36], [37] (Fig. 2H). *ccdc80-l1* expression in adaxial cells persisted at 36 hpf and 48 hpf (Fig. 2I, 2K). At the same stages *ccdc80-l1* is also expressed in the caudal vein plexus region (Fig. 2I, 2J, 2L).

### *ccdc80-l1* knocked-down embryos displayed impaired motility

To determine the functional role of *ccdc80-l1* during zebrafish development, we specifically knocked it down by means of the injection of an antisense oligonucleotide morpholino (*ccdc80-l1*-MO, Gene Tools) designed against the start site of the transcript. In all the experiments, *ccdc80-l1*-MO-injected embryos (morphants) were compared to embryos at the same developmental stage, injected with the same amount of a control MO (ctrl-MO). For the *in vivo* test of the efficiency of *ccdc80-l1*-MO, 425 pg/embryo of the pCS2+-*ccdc80-l1*-GFP sensor plasmid has been injected alone or with 12 ng/embryo of *ccdc80-l1*-MO. The presence/absence of the GFP signal has been monitored under a fluorescent microscope starting from somitogenesis up to 48 hpf (Fig. S2). 70% of the embryos (N = 20) injected with the sensor plasmid alone displayed fluorescence. This percentage decreased to 51% when the plasmid was co-injected with *ccdc80-l1*-MO (N = 93), indicating that the morpholino specifically bound to its target region. The efficiency of *ccdc80-l1* loss-of-function was not so striking, as demonstated by the low percentage of embryos with GFP decreasement and by the high amount of morpholino we had to inject to obtain a phenotype (8 and 12 ng/embryo of *ccdc80-l1*-MO). Therefore, we designed a second morpholino against the splice site (*ccdc80-l1*-splice-MO) to confirm the specificity of *ccdc80-l1*-loss-of-function. Embryos injected with this second morpholino, still exhibited motility issues as *ccdc80-l1*-MO-injected embryos did (data not shown). In particular, all the knocked-down embryos showed no severe body plan alteration when observed *in vivo*, indicating the proper progression of early developmental processes such as gastrulation and segmentation [38], [39], [40], [41]. Moreover, we observed that morphants displayed physiological body contractions upon dechorionation at 24 hpf [42]. However, at 48 hpf, almost 80% of the morphant embryos (N = 37) presented abnormal escaping behavior after tactile stimulation, often resulting in body contractions on the spot or circling behavior (Video S2) rather than a fast escape in the opposite direction to the stimulus (Video S1). The same phenotype was observed also at 5 days post fertilization (5 dpf, data not shown). These results indicated that *ccdc80-l1* loss-of-function affects the swimming behavior of zebrafish embryos and larvae. We were also able to rescue the *ccdc80-l1*-loss-of-function phenotype by means of the injection of the homolog *ccdc80*-full-length transcript. In fact, despite we demonstrated that *ccdc80*-loss-of-function did not affect axonal pathfinding (data not shown), the high degree of conservation between the two homologs allowed rescue of motility (only 42% of rescued embryos presented motility issues in comparison to the nearly 80% of *ccdc80-l1*-MO injected embryos, N = 63).

### *ccdc80-l1* loss of function does not affect somitogenesis nor muscle pioneers and adaxial cells formation

**Figure 3:**
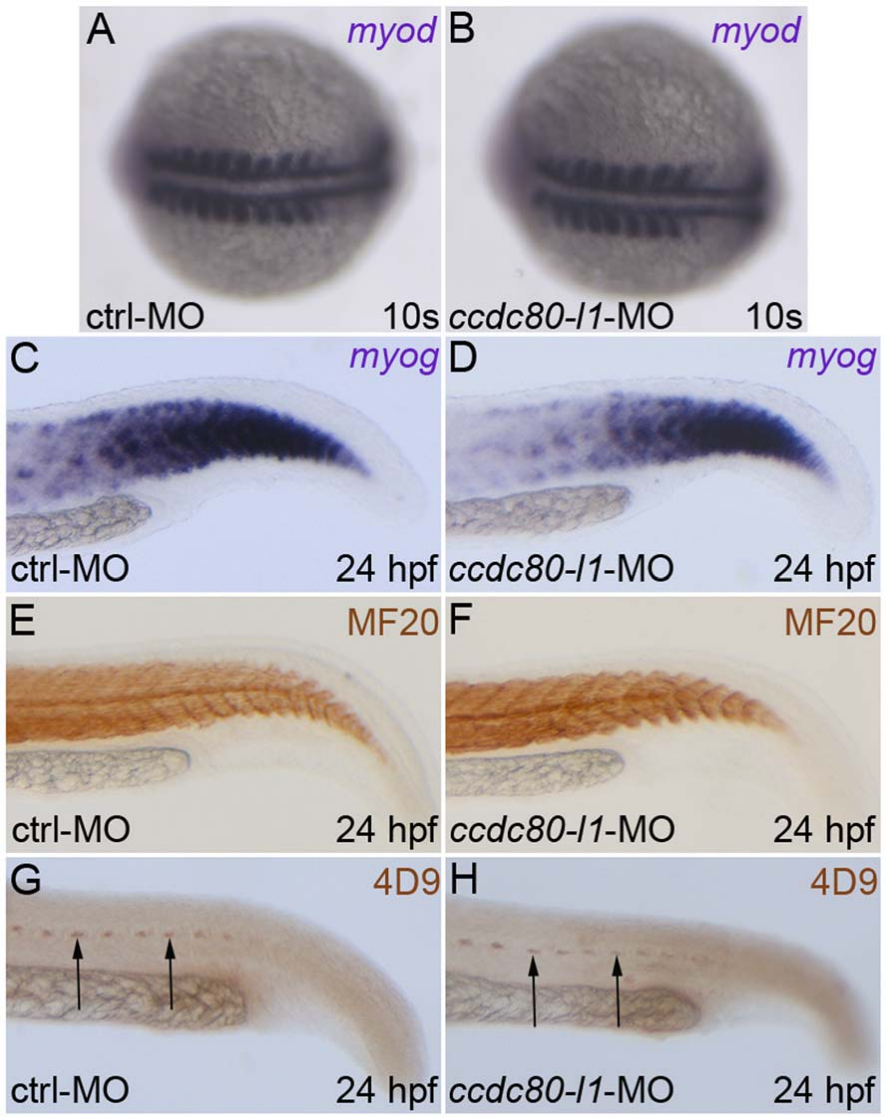
Analysis of myogenic markers expression and muscle pioneers in *ccdc80-l1* morphant embryos. *(A–D)* The myogenic markers *myod (A, B)* and *myog (C, D)* were correctly expressed both in control and morphants embryos at 10 s stage *(A, B)* and 24 hpf *(C, D)*, respectively. *(E, F)* The MF20 antibody staining showed that both slow and fast twitch fibers were correctly formed and distributed in control and in knocked-down embryos at 24 hpf. *(G, H)* At the same developmental stage, muscle pioneers resulted unaffected after *ccdc80-l1* loss-of-function, as shown by the labeling with 4D9 antibody (anti-engrailed) (arrows). *(A, B)* Dorsal views, anterior is left; *(C–H)* lateral views of the tails, dorsal is up and anterior is left.

To assess whether the phenotype displayed by morphants was due to the impairment of musculature, we examined somitogenesis and myogenesis markers. The expression of *myod* and *myog* [39], [43] was not altered in *ccdc80-l1*-MO-injected embryos (Fig. 3A–D). Moreover, the expression of *smyhc1*, a marker of slow-twitch fibers [44], was unaffected as well, notwithstanding the strong expression of *ccdc80-l1* in adaxial cells and muscle pioneers, from which slow fibers develop [9] (Fig. S3A, S3B). In addition, at 24 hpf, myofibers were correctly organized and distributed as shown by the immunohistochemistry with anti-sarcomeric MF20 antibody [45] (Fig. 3E, 3F). Also muscle pioneers, labeled with 4D9 anti-engrailed antibody [36], [37] were correctly formed in *ccdc80-l1* morphants (Fig. 3G–H). These results led us to exclude that defects of adaxial cells, muscle pioneers or body musculature formation were responsible for motility issues observed in *ccdc80-l1* knocked-down embryos.

### Proper pathfinding and branching of axons are affected in *ccdc80-l1*-MO-injected embryos

**Figure 4:**
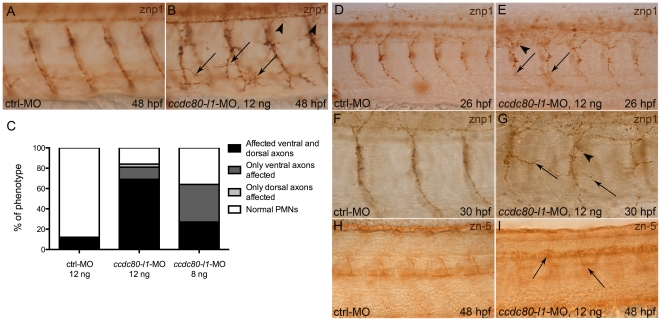
Analysis of motoneurons morphology by means of znp1- and zn-5-immunohistochemistry. *(A, B)* At 48 hpf, using 12 ng/embryo of morpholino, both ventral (arrows) and dorsal axons (arrowheads) were mis-orientated and over-branched in morphants *(B)* in comparison to control embryos *(A)*. *(C)* Statistical analysis showing the percentages of the different phenotypes (affected ventral axons, dorsal axons or both) occurring in control embryos and in morphants, when different doses of *ccdc80-l1*-MO were injected (12 ng/embryo and 8 ng/embryo). Using a lower dose of morpholino (8 ng/embryo), we observed that in a significant percentage of embryos only ventral axons were defective. *(D–G)* Immunohistochemistry performed at 26 hpf *(D, E)* and 30 hpf *(F, G)* confirmed that loss-of-*ccdc80-l1*-function affects both CaPs (arrows) and MiPs (arrowheads) axonal migration. *(H, I)* The same analysis performed at 48 hpf using zn-5 antibody revealed that also SMNs axonal migration is impaired in morphants (arrows in *I*) in comparison to control embryos *(H). (A, B; D–I)* Lateral flat-mount preparation was applied for a better visualization of the motoneurons. Lateral views of the trunk region overhanging the yolk extension, dorsal is up and anterior is left.

Segmentally repeated motoneurons connect nervous system to somites, as their growth cones exit the spinal cord during embryogenesis and migrate to their appropriate muscle targets, allowing movement [3], [5]. Due to motility impairment of *ccdc80-l1* morphants, we investigated the morphology of motoneurons performing immunohistochemistry with znp1 (*syt2b*) antibody [46]. For all embryos, we analyzed the trunk region overhanging the yolk extension; defects in at least three motoneurons were enough to consider the embryo as affected. At 48 hpf, in the 84% (N = 33) of morphants injected with 12 ng/embryo of morpholino, axonal pathfinding resulted impaired. 60% of morphants displayed an overall disorganization of both dorsal and ventral motoneurons, that resulted mis-orientated and over-branched (Fig. 4A, 4B). In the 9% of embryos, these defects were observed together with an opposite phenomenon, axonal stalling. In the 12% of morphants only ventral axons resulted mis-orientated and over-branched, whereas in the 3% only the dorsal ones were affected. This phenotype was dose-dependent: when 8 ng/embryo of morpholino were used, a lower percentage of embryos resulted affected (64%, N = 35). Interestingly, at this concentration, only 27% of the knocked-down embryos displayed both ventral and dorsal defective axons, whereas in the 37% of morphants the same defects were detectable in the ventral motoneurons solely (Fig. 4C). Dorsal axons alone were never affected (Table 2 and Fig. S4). Thus, a striking reduction of Ccdc80-l1 protein amount led to the affection of both ventral and dorsal motoneurons, whereas a lower dose of morpholino is sufficient for ventral axons migration impairment. In order to discriminate whereas loss-of-*ccdc80-l1*-function impaired PMNs or SMNs, we analyzed the phenotype of morphants also at 26 hpf and 30 hpf, by the time SMNs have just begun extending axons [47], so most of znp1 labeling correspond to PMNs. At this stages, we replicated the same phenotype observed at 48 hpf (Fig. 4D–G and Table 3). Furthermore, by performing an immunohistochemistry at 48 hpf using the specific antibody for SMNs (zn-5, anti-*alcama*) [47], we observed that also SMNs axons seems to be affected after loss-of-*ccdc80-l1*-function (Fig. 4H, 4I). Thus, the analysis of the motoneuronal patterning in morphant embryos revealed the lack of proper guidance toward muscle targets, suggesting *ccdc80-l1* involvement in axonal pathfinding of both PMNs and SMNs.

**Table 2:** The phenotype of *ccdc80-l1*-MO-injected embryos is dose-dependent.

Dose/type of morpholino	Total percentage of affected embryos (N)	Alteration of both ventral and dorsal axons	Only defective ventral axons	Only defective dorsal axons
ctrl-MO 12 ng	12% (N = 25)	12%	0%	0%
*ccdc80-l1*-MO 12 ng	84% (N = 33)	69%	12%	3%
*ccdc80-l1*-MO 8 ng	64% (N = 35)	27%	37%	0%

The percentage of embryos displaying axonal defects decreased from 84% to 64% when a lower dose of morpholino was used. Both ventral and dorsal axonal pathfinding resulted impaired in the 69% of affected embryos when 12 ng/embryo of morpholino were used. After the injection of the lower dose of *ccdc80-l1*-MO (8 ng/embryo), 27% of affected embryos showed alteration of both ventral and dorsal axons, whereas the 37% displayed only ventral defective axons and dorsal axons alone were never affected.

**Table 3:** Loss-of-*ccdc80-l1*-function impairs PMNs axonal migration.

Developmental stage/dose of morpholino	Total percentage of affected embryos (N)	Alteration of both CaPs and MiPs	Only CaPs affected	Only MiPs affected
26 hpf/*ccdc80-l1*-MO 12 ng	54,5% (N = 33)	40%	11%	3,5%
30 hpf/*ccdc80-l1*-MO 12 ng	62% (N = 35)	33%	24,5%	4,5%

Embryos injected with 12 ng/embryo of *ccdc80-l1*-MO were observed also at 26 hpf and 30 hpf. At these stages, affected embryos were 54,5% and 62%, respectively. The percentages of the different phenotypes are listed.

### *ccdc80-l1* expression is positively regulated by the Hedgehog pathway

**Figure 5:**
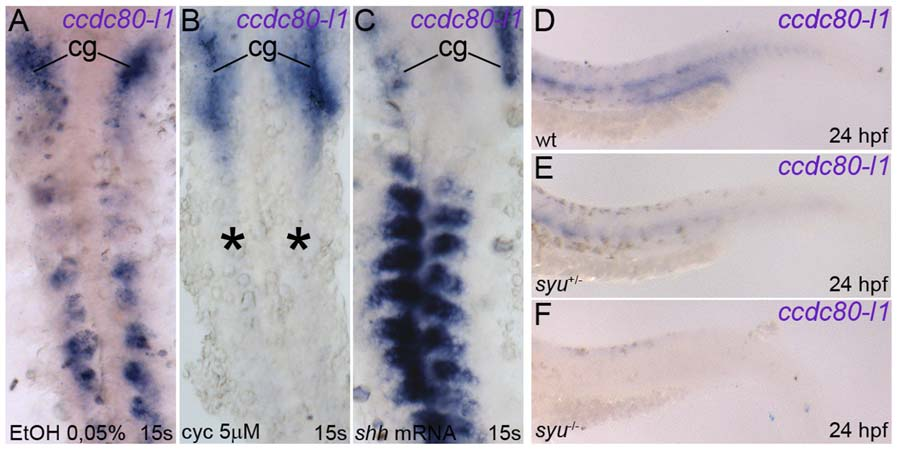
*ccdc80-l1* is positively regulated by *shh*. *(A–C) ccdc80-l1* expression in somites and myoseptum resulted strongly inhibited in embryos treated with 5 µM cyclopamine (asterisks in *B*), in comparison to control embryos at the same developmental stage *(A)*. By converse, over-expression of *shh* led to an up-regulation of *ccdc80-l1* in muscular territories *(C)*. Expression in cranial ganglia (cg) was never perturbed. *(D–F) ccdc80-l1* resulted slightly down-regulated in the muscles of heterozygous *syu* ^+/−^ mutants *(E)* in comparison to wild type siblings *(D)*. A strikingly down-regulation was observed in homozygous *syu* ^−/−^ mutants *(F)*. *(A–C)* Dorsal flat-mount preparations, anterior is up. *(D–F)* Lateral views of the tails, anterior is left.

Both muscle and motoneurons induction is finely regulated by levels and range of *shh* expression [9], [48], [49]. Due to *ccdc80-l1* expression in adaxial cells and muscle pioneers, we decided to investigate the existence of a *ccdc80-l1* up-stream regulation Hedgehog-mediated. We modulated *shh* activity by exposing embryos to cyclopamine, that inhibits the *Hedgehog* transducer Smoothened (smo) [9], [50]. To avoid the complete loss of the territories in which *ccdc80-l1* is expressed, we chose a concentration of cyclopamine (5 µM) by which muscle pioneers and adaxial cells-derived slow fibers are unaffected, as already described [9] and as we demonstrated by the proper expression of their markers engrailed, *myod* and *smyhc1* respectively. (Fig. S5). A striking down-regulation of *ccdc80-l1* expression was observed in 72% of the treated embryos in comparison to controls (N = 32) (Fig. 5A, 5B). Interestingly, this down-regulation was detectable only at the level of myoseptum and somites, whereas the cephalic territories in which *ccdc80-l1* is expressed were not involved. A similar down-regulation was observed in *syu* mutants, carriers of a deletion in the gene *sonic-you* encoding for *shh* (Fig. 5D–F) [16]. *ccdc80-l1* signal in adaxial cells was extremely weak or absent in the 35% of mutants, and slightly down-regulated in the 40% of observed embryos (N = 20). Moreover, the overexpression of *shh* by means of the injection of the full-length transcript (300 pg/embryo), led to the opposite phenotype with an increasing of *ccdc80-l1* expression in the somites of the 71% of the injected embryos (N = 31) (Fig. 5C). On the contrary, *ccdc80-l1* loss-of-function did not affect *shh* expression (Fig. S6). Therefore, these findings suggest that *ccdc80-l1* is a down-stream target of the Hedgehog pathway.

## Discussion

The genetic program underlying axon guidance is not completely defined. Adaxial cells and muscle pioneers are both involved in axonal outgrowth and pathfinding [7], [17], even if little is known about the specific proteins and molecular mechanisms acting in this process. *ccdc80-l1*, the novel gene we recently identified in zebrafish, is expressed during embryonic development in muscle pioneers and adaxial cells. Ccdc80-l1 was identified, together with its homolog Ccdc80, on the basis of its high aminoacid identity with human CCDC80. However, zebrafish *ccdc80* and *ccdc80-l1* do not share the same expression pattern and seems to play different roles. In fact, only *ccdc80-l1*-MO-injected embryos displayed an abnormal escaping behavior after tactile stimulation at 48 hpf. Both musculature and nervous system are responsible for embryonic motility and touch response and are the basis of spontaneous motor output that occurs in the developing zebrafish embryo ever since 18 hpf [2]. Nevertheless, musculature defects were unlikely the basis of the observed phenotype. Indeed, there was no difference between the expression pattern of myogenic markers in morphants and control embryos. Moreover, muscle fibers resulted correctly formed and distributed by the end of somitogenesis. The territories in which *ccdc80-l1* is expressed were unaffected as well: in fact, adaxial cells and muscle pioneers showed no defects. These findings revealed that *ccdc80-l1* function is not necessary for the specification and further differentiation of myogenic cell populations, suggesting that the motility issues displayed by morphants at 48 hpf could be due to an impairment of neuronal development.

The analysis of motoneuronal development in morphant embryos revealed that *ccdc80-l1* plays a role in motoneurons axonal pathfinding. In fact, *ccdc80-l1* loss-of-function did not prevent the formation of PMNs and axon projection, but led to an overall disorganization of PMNs. CaPs and MiPs resulted over-branched in a high percentage of embryos, whereas a smaller fraction of morphants displayed also the simultaneous presence of its opposite phenomenon, axonal stalling. Axonal over-branching and stalling were detected in the CaPs solely in a significant percentage of embryos, especially when a lower dose of morpholino was used. Moreover, the impairment of axonal migration was more severe in CaPs then in MiPs, even when both PMNs were affected simultaneously. These data suggest that *ccdc80-l1* may have a differential role as regards the development of CaPs and MiPs. This is consistent with the asymmetric distribution of *ccdc80-l1* transcript in the somites: indeed, the *ccdc80-l1* transcript is present in the ventral portion of somites, innervated by CaPs, and not in their dorsal portion, innervated by MiPs. The same motility issues displayed at 48 hpf were observed also at 5 dpf, when secondary motoneurons are already formed. Therefore, *ccdc80-l1* plays a role also in guidance of SMNs, as shown by the mis-expression of their marker zn-5. This is not surprising, as the growth cones of SMNs require the axons of PMNs for proper pathfinding [6]. We concluded that the *ccdc80-l1* loss-of-function prevents the proper development of the peripheral nervous system, that lacks a proper guidance toward muscle target: axons do not fallow a single direction-pathway but stall or extend towards any direction, leading to an over-branched and non-functional nervous network. Hence, embryos are able to move and to respond to tactile stimuli, but the coordination of muscle contractions is impaired, and motor behavior is affected.

Axon outgrowth is influenced by many factors, for instance different molecules (netrins, semaphorins, slits) with chemotropic functions (reviewed in [51]) and components of the extracellular matrix (ECM) [52], [53]. In fact, the growth cone shares many features with the motile structures of migrating cells, including actin polymerization at the leading edge, dynamic interactions between cell-surface adhesion receptors and components of the extracellular matrix (ECM), and generation of traction forces in the cytoskeleton applied to ECM through adhesion sites [54], [55]. In *ccdc80-l1* knocked-down embryos, the growth cones are still able to exit the spinal cord and reach the muscle pioneers along the common pathway. Moreover, axonal extensions developed without altering the target choice: in fact, CaPs and MiPs still project their axons ventrally and dorsally, respectively. These data are consistent with the proper development of muscle pioneers, which provide a choice point for motor growth cones. However, further defects occur during axon pathfinding. It has been recently reported that DRO1/CCDC80 is a Golgi-associated-protein [56]. Moreover, the *in silico* prediction of the Ccdc80 protein structure (String 9.0) suggests its interaction with fibronectin, a component of the ECM. If this is the case also for its homolog *ccdc80-l1*, its loss-of-function might interfere with the proper axon migration by influencing the secretion of guidance molecules or by altering interactions with ECM proteins such as fibronectin. Further analysis on the predicted Ccdc80-l1 protein sequence and its interaction with other proteins will be necessary to understand the molecular process underlying *ccdc80-l1* functioning. Moreover, investigation on possible targets is still needed. For instance, it is to explore the possibility of an interaction with the semaphorin and netrin families, both involved in attracting and/or repelling growth cones from a variety of organisms [13], [19]. Nevertheless, our results provide further insights into motoneurons development, a complex mechanism that requires the action of several different molecules. Moreover, we suggest that *ccdc80-l1* may act as a down-stream effector of *shh*. The Hedgehog family consists of secreted morphogens fundamental for both axon guidance and formation of adaxial cells and muscle pioneers [9], [57]. The Hedgehog signaling is known to play a pivotal role in the specification of both primary and secondary motoneurons [14], [49]. Indeed, mutants for different molecules involved in this pathway displayed axonal defects, including random axonal migration or stalling [15], [16]. PMNs target choice was never impaired after *ccdc80-l1* loss-of-function, still axonal migration resulted aberrant. Furthermore, *ccdc80-l1* expression resulted strikingly down-regulated after exposure to 5 µM of cyclopamine and up-regulated after over-expression of *shh*. This modulation was observed only in muscles and not in other territories in which *s-ccc80* is expressed (cranial ganglia and dorsal dermis). These findings strongly suggest the existence of a specific regulation Hedgehog-mediated of *ccdc80-l1,* as regards its function in motoneuronal development. Moreover, these findings may shed light on the involvement of the Hedgehog pathway in this process.

## Supporting Information

Figure S1 **Alignment among human CCDC80 and the three zebrafish homologs.** * = identical aminoacids; :  = conservative substitution; .  = non-conservative substitution.
(TIF)

Figure S2 ***ccdc80-l1*** **morpholino is capable to inhibit the expression of the fluorescent protein GFP.** This assay was performed in order to verify the *in vivo* efficiency of *ccdc80-l1*-MO. *(A, B)* In the 70% of embryos injected with the *ccdc80-l1*-GFP sensor plasmid, the presence of fluorescent GFP signal was detected (N = 20). *(C, D)* When the plasmid was injected together with the morpholino, the transcription of GFP protein was inhibited and the percentage of fluorescent embryos decreased to 51% (N = 93). In *A* and *C* embryos are visualized under normal light, in *B* and *D* under fluorescent light.
(TIF)

Figure S3 **The expression pattern of the slow-myosin marker** ***smyhc1*** **is unaffected in** ***ccdc80-l1*** **knocked-down embryos.** *(A, B)* Loss-of-*ccdc80-l1-*function did not perturb the expression of *smyhc1*, as morphant embryos *(B)* are indistinguishable from control embryos *(A).* Lateral views of the tails, dorsal is up, anterior is left.
(TIF)

Figure S4 **Statistical analysis of three distinct defects observed after loss-of-** ***ccdc80-l1*** **-function.** *(A–C)* The graphics show the occurrence of three axonal migration defects in control embryos and morphants when two doses of *ccdc80-l1*-MO are used: both dorsal and ventral defective axons *(A)*, only ventral defective axons *(B)* and only dorsal defective axons *(C)*. The last phenotype was not statistically significant. *** p<0.001 vs ctrl-MO. * p<0.05 vs ctrl-MO.
(TIF)

Figure S5 **Muscle pioneers and adaxial cells are present after 5 µM cyclopamine treatment.** *(A, B)* Labeling with 4D9 antibody (anti-engrailed) showed that muscle pioneers are not missing after pharmacological inhibition of the Hedgehog pathway (arrows). *(C–F)* Also adaxial cells are still present, as shown by the expression of the markers *myod (C, D)* and *smyhc1 (E, F)*. *(A, B)* Lateral views, dorsal is up. *(C–F)* Dorsal views, anterior is left.
(TIF)

Figure S6 ***shh*** **expression is not perturbed by loss-of-** ***ccdc80-l1*** **-function.** *(A, B) shh* resulted correctly expressed both in control embryos *(A)* and in morphants *(B)*. *(A, B)* Dorsal views, anterior is left.
(TIF)

Video S1 **Control embryos displayed standard motor behavior.** After tactile stimulation, control embryos fast escaped in the opposite direction of the stimulus.
(AVI)

Video S2 **The motility of morphant embryos is impaired.** When morphants were stimulated, abnormal escape was observed, also resulting in circling behavior.
(AVI)
