# Supplementary figures and images for: ccdc80-l1 Is Involved in Axon Pathfinding of Zebrafish Motoneurons
Source: PLoS One. 2012 Feb 22;7(2):e31851. doi: 10.1371/journal.pone.0031851 (PMC3285184; doi:10.1371/journal.pone.0031851)

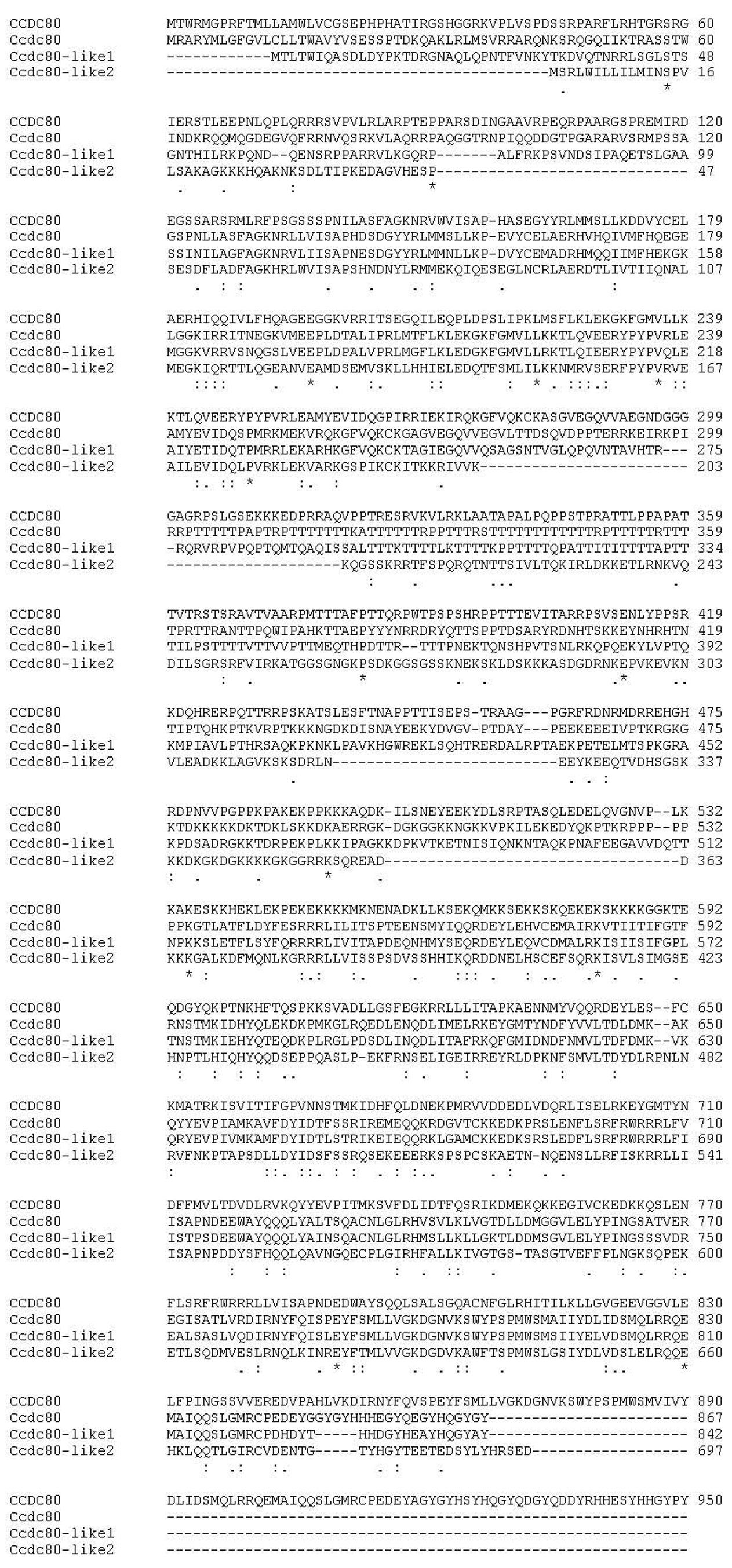

Supplement: Figure S1 — Alignment among human CCDC80 and the three zebrafish homologs. * = identical aminoacids; : = conservative substitution; . = non-conservative substitution. (TIF) [file pone.0031851.s001.tif]

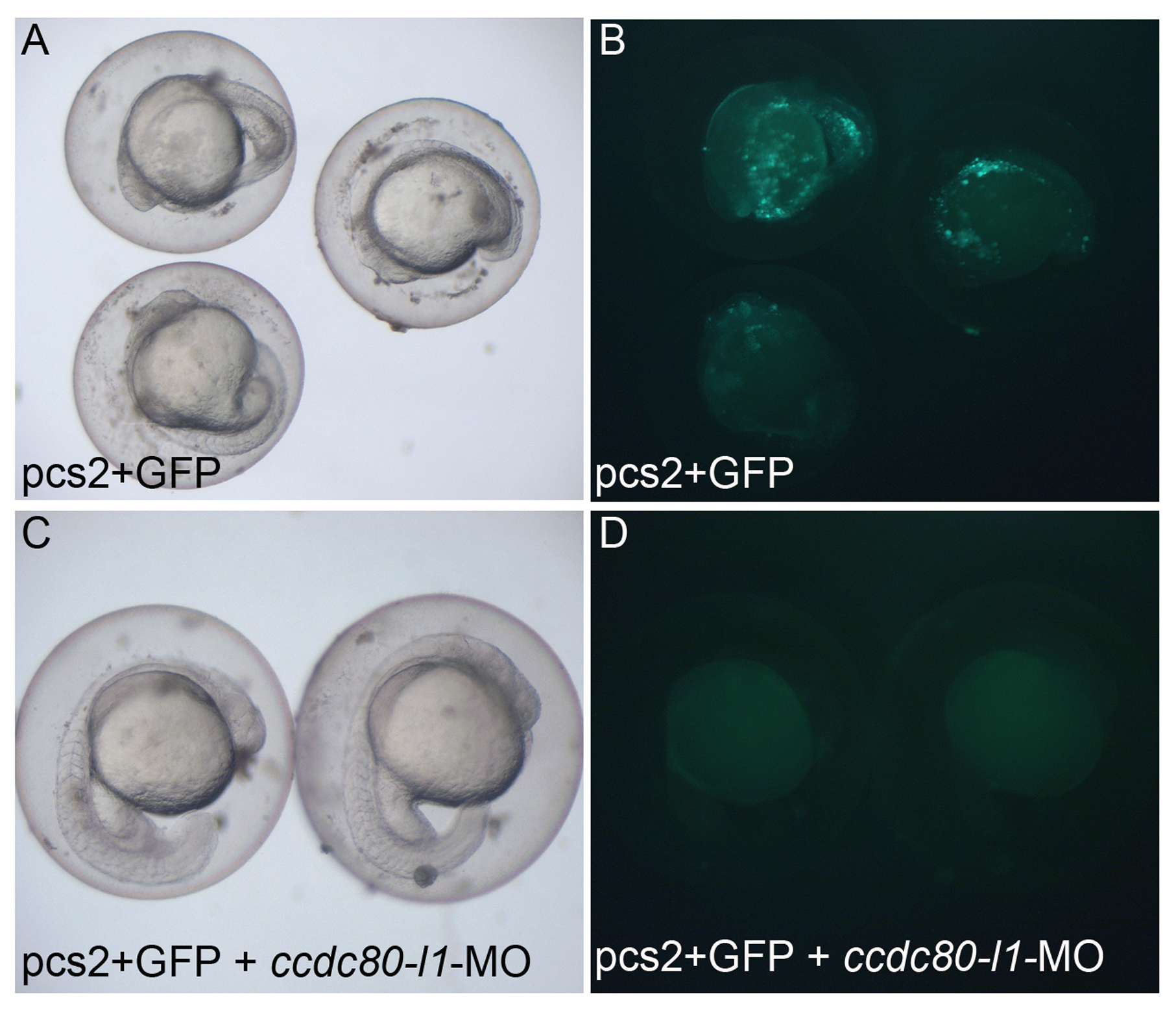

Supplement: Figure S2 — ccdc80-l1 morpholino is capable to inhibit the expression of the fluorescent protein GFP. This assay was performed in order to verify the in vivo efficiency of ccdc80-l1-MO. (A, B) In the 70% of embryos injected with the ccdc80-l1-GFP sensor plasmid, the presence of fluorescent GFP signal was detected (N = 20). (C, D) When the plasmid was injected together with the morpholino, the transcription of GFP protein was inhibited and the percentage of fluorescent embryos decreased to 51% (N = 93). In A and C embryos are visualized under normal light, in B and D under fluorescent light. (TIF) [file pone.0031851.s002.tif]

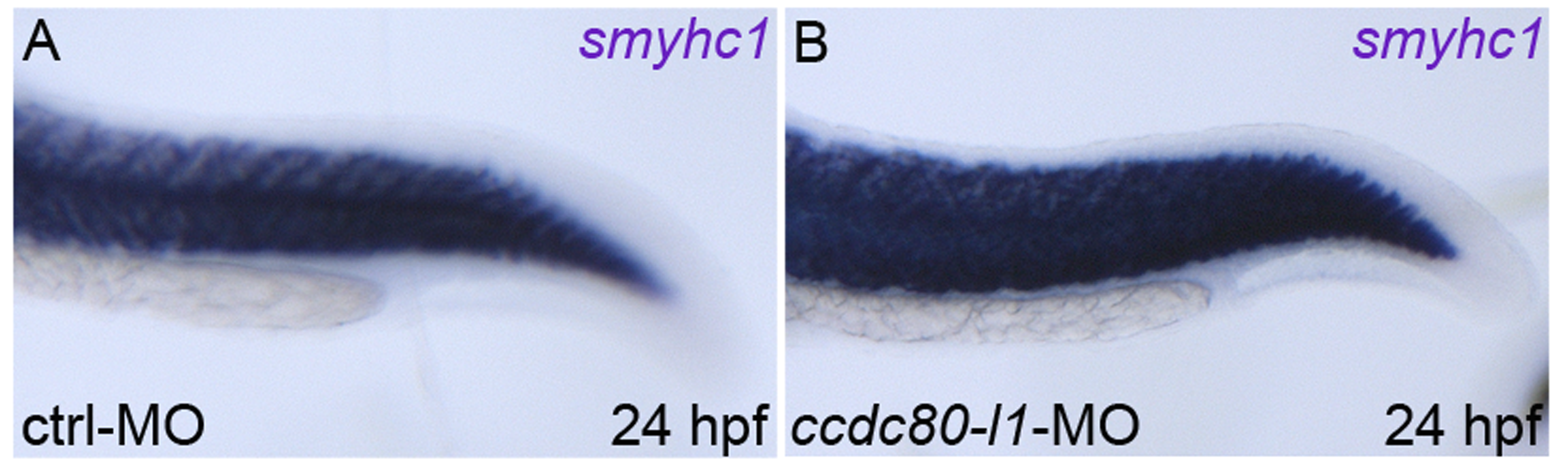

Supplement: Figure S3 — The expression pattern of the slow-myosin marker smyhc1 is unaffected in ccdc80-l1 knocked-down embryos. (A, B) Loss-of-ccdc80-l1-function did not perturb the expression of smyhc1, as morphant embryos (B) are indistinguishable from control embryos (A). Lateral views of the tails, dorsal is up, anterior is left. (TIF) [file pone.0031851.s003.tif]

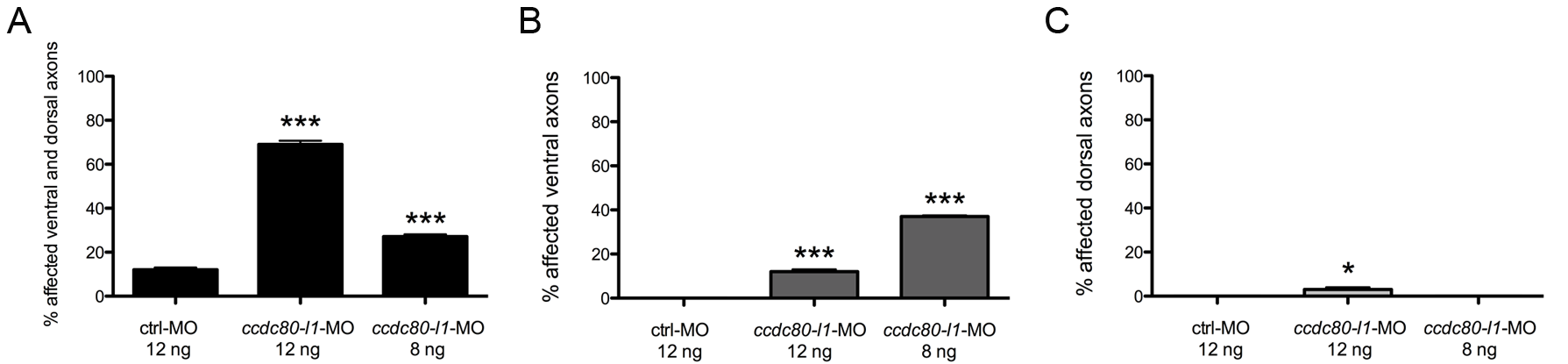

Supplement: Figure S4 — Statistical analysis of three distinct defects observed after loss-of- ccdc80-l1 -function. (A–C) The graphics show the occurrence of three axonal migration defects in control embryos and morphants when two doses of ccdc80-l1-MO are used: both dorsal and ventral defective axons (A), only ventral defective axons (B) and only dorsal defective axons (C). The last phenotype was not statistically significant. *** p<0.001 vs ctrl-MO. * p<0.05 vs ctrl-MO. (TIF) [file pone.0031851.s004.tif]

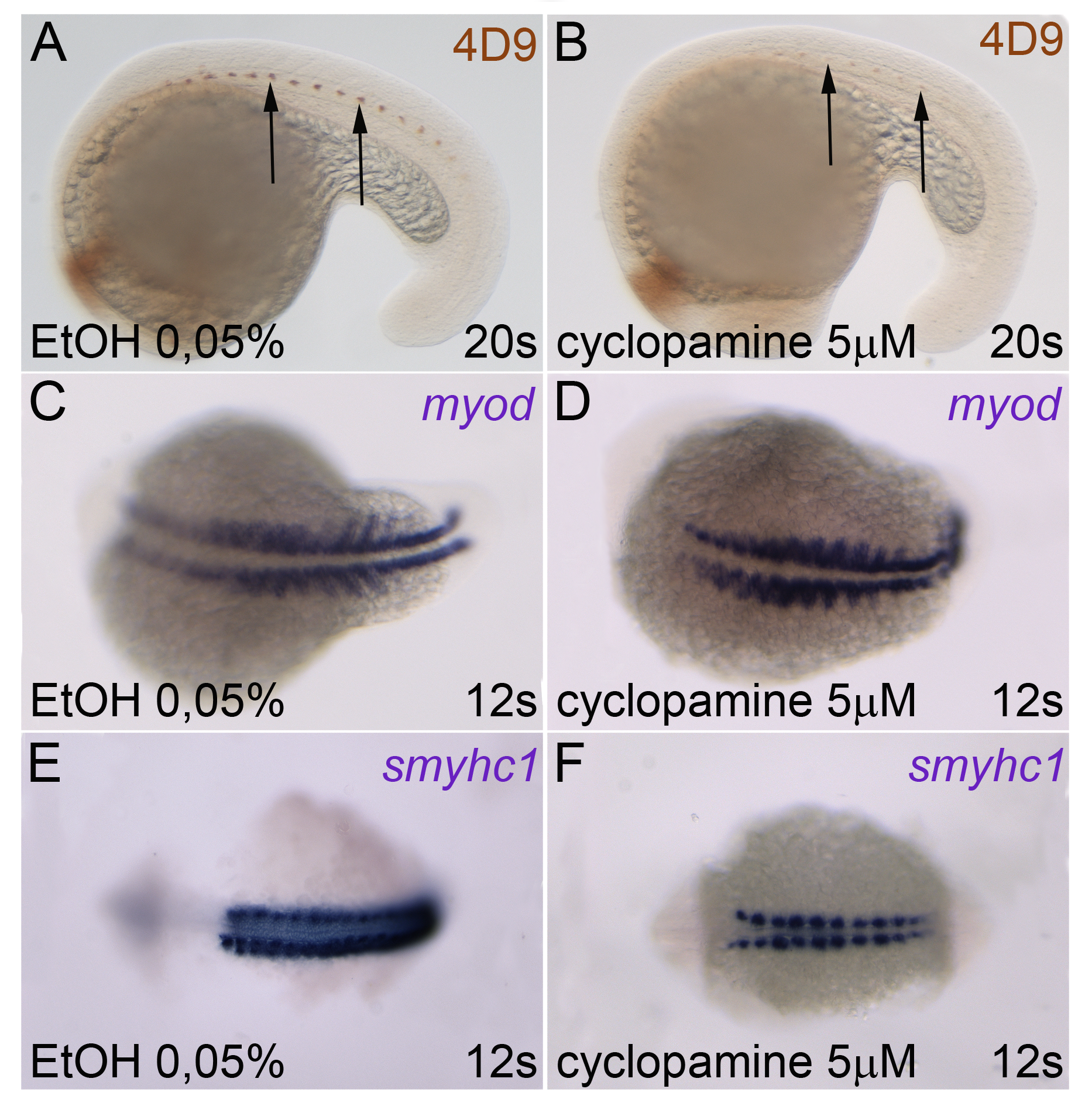

Supplement: Figure S5 — Muscle pioneers and adaxial cells are present after 5 µM cyclopamine treatment. (A, B) Labeling with 4D9 antibody (anti-engrailed) showed that muscle pioneers are not missing after pharmacological inhibition of the Hedgehog pathway (arrows). (C–F) Also adaxial cells are still present, as shown by the expression of the markers myod (C, D) and smyhc1 (E, F). (A, B) Lateral views, dorsal is up. (C–F) Dorsal views, anterior is left. (TIF) [file pone.0031851.s005.tif]

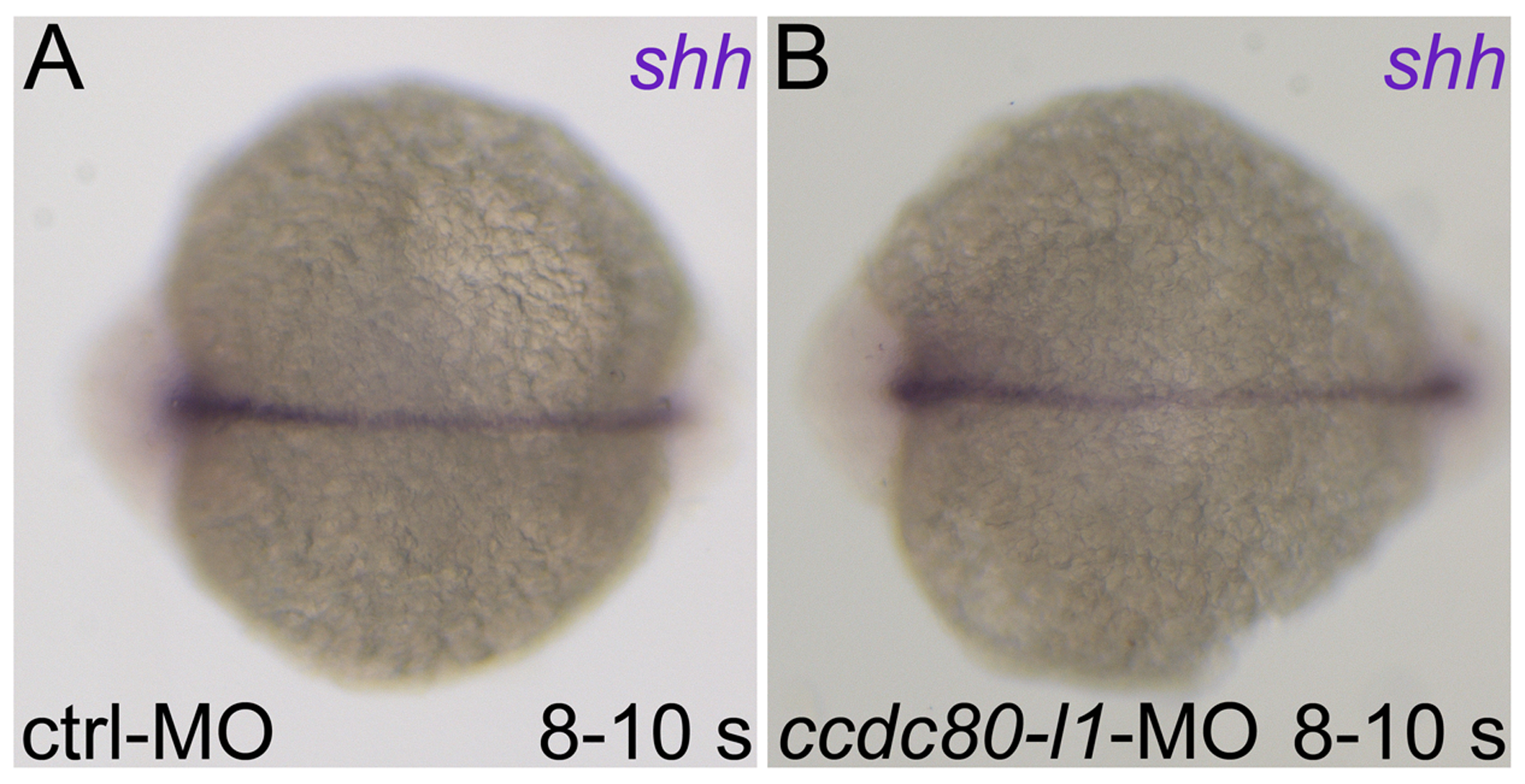

Supplement: Figure S6 — shh expression is not perturbed by loss-of- ccdc80-l1 -function. (A, B) shh resulted correctly expressed both in control embryos (A) and in morphants (B). (A, B) Dorsal views, anterior is left. (TIF) [file pone.0031851.s006.tif]
